# Critical race theory as a bridge in science training: the California State University, Northridge BUILD PODER program

**DOI:** 10.1186/s12919-017-0089-2

**Published:** 2017-12-04

**Authors:** Carrie L. Saetermoe, Gabriela Chavira, Crist S. Khachikian, David Boyns, Beverly Cabello

**Affiliations:** 1 Department of Psychology, BUILD Co-PIs, CSUN, 18111 Nordhoff Street, Northridge, CA 91330-8255 USA; 2 CSUN Office of Research and Graduate Studies, BUILD Co-PI, Northridge, CA 91330-8222 USA; 3 Department of Sociology and the Center for Assessment, Research, and Evaluation (CARE), Northridge, CA 91330-8352 USA; 4 Department of Educational Psychology and Counseling, Co-Director, Center for Assessment, Research, and Evaluation (CARE), Northridge, CA 91330-8265 USA

## Abstract

**Background and purpose:**

Unconscious bias and explicit forms of discrimination continue to pervade academic institutions. Multicultural and diversity training activities have not been sufficient in making structural and social changes leading to equity, therefore, a new form of critical consciousness is needed to train diverse scientists with new research questions, methods, and perspectives. The purpose of this paper is to describe Building Infrastructure Leading to Diversity (BUILD); Promoting Opportunities for Diversity in Education and Research (PODER), which is an undergraduate biomedical research training program based on transformative framework rooted in Critical Race Theory (CRT).

**Key highlights:**

By employing a CRT-informed curriculum and training in BUILD PODER, students are empowered not only to gain access but also to thrive in graduate programs and beyond. Poder means “power” or “to be able to” in Spanish. Essentially, we are “building power” using students’ strengths and empowering them as learners. The new curriculum helps students understand institutional policies and practices that may prevent them from persisting in higher education, learn to become their own advocates, and successfully confront social barriers and instances of inequities and discrimination. To challenge these barriers and sustain campus changes in support of students, BUILD PODER works toward changing campus culture and research mentoring relationships. By joining with ongoing university structures such as the state university Graduation Initiative, we include CRT tenets into the campus dialogue and stimulate campus-wide discussions around institutional change. Strong ties with five community college partners also enrich BUILD PODER’s student body and strengthen mentor diversity. Preliminary evaluation data suggest that BUILD PODER’s program has enhanced the racial/ethnic consciousness of the campus community, is effective in encouraging more egalitarian and respectful faculty-student relationships, and is a rigorous program of biomedical research training that supports students as they achieve their goals.

**Implications:**

Biomedical research programs may benefit from a reanalysis of the fit between current training programs and student strengths. By incorporating the voices of talented youth, drawing upon their native strengths, we will generate a new science that links biomedical research to community health and social justice, generating progress toward health equity through a promising new generation of scholars.

## Background and context

### Introduction to critical race theory and BUILD PODER

California State University, Northridge’s BUILD PODER program (Building Infrastructure Leading to Diversity; Promoting Opportunities for Diversity in Education and Research), funded by the National Institutes of Health, is diversifying the biomedical workforce by reframing and redesigning institutional practices, undergraduate biomedical research training, and research mentoring approaches through the lens of Critical Race Theory (CRT) [1–3]. In BUILD PODER, students are empowered to not only gain access but also thrive in graduate programs and beyond, employing a CRT-informed curriculum and training. *Poder* means “power” or “to be able to” in Spanish. Essentially, we are “building power”—empowering students. The curriculum helps students understand institutional policies and practices that may prevent them from persisting in higher education, learn to become their own advocates, and successfully confront social barriers and instances of inequities and discrimination.

Critical Race Theory has its roots in the 1960s Civil Rights and 1970s Critical Legal Studies movements and “critically interrogate[s] how the law reproduces, reifies, and normalizes racism in society” [2]. There are five central tenets of CRT that form its basic perspective, pedagogy and research methodology: (1) the centrality of race and racism; (2) the challenge to dominant ideology; (3) an interdisciplinary perspective; (4) the importance of students’ experiential knowledge; and (5) a commitment to social justice [3, 4].

Critical Race Theory provides educators and researchers with a framework to challenge the racist historical and institutional roots of educational inequality that persist today [5–15]. Social reproduction of roles [16] reifies a power structure that invites Students of Color to feel like interlopers, particularly at Predominantly White Institutions (PWI) or universities originally PWI. These power structures are maintained by (1) “race neutral” admissions and colorblindness [17, 18]; (2) majoritarian narratives of meritocracy and deficit thinking [17, 19]; (3) “the social construction of merit” as rigidly defined [20]; (4) programmatic interest convergence that benefits Whites more than people of color, such as affirmative action [21–23]; and (5) the use of “diversity” as a commodity in university marketing tools [15, 24–26].

Through the lens of CRT, BUILD PODER is working to interrupt the majoritarian narrative by building a community of biomedical scholars, students, faculty, and administrators equipped with the tools to develop more egalitarian, respectful structures and relationships in biomedical research and mentoring by addressing, for example, unconscious bias, White privilege, microaggressions, stereotype threat, structural racism, historical trauma, and the influence of racism on science. Through education and activities, students and mentors also better understand the intersectionality of race with gender, ability, sexual orientation, as marginalized groups [27–30]. BUILD PODER biomedical undergraduate students are prepared with a rigorous, advanced research curriculum and lab work, professional development, and a sense of meaning that allows them to contextualize their life’s work. BUILD PODER encourages students to maintain their native identity by honoring their family’s heritage [9, 31, 32] and by preparing them to take on STEM research topics that integrate their own experiential knowledge [20] and their social and cultural capital [15, 18, 33] so that they can become the highly skilled, socially conscious scientists of the future who will take us significantly closer to health equity, a central focus of BUILD PODER that is linked to social justice and health as a human right.

### Linking biomedical research to social justice: CRT, health equity, and praxis

“CRT practice attends to the voice of the marginalized by placing it in social context, and translating personal pain into a social justice agenda at the direct practice level” [16]. The primary link between social justice and research in BUILD PODER focuses on health disparities and health equity. Despite concerted efforts to reduce mortality and morbidity, racial/ethnic health disparities remain in nearly every area of health [34] including cancer, cardiovascular disease, diabetes, HIV/AIDS, and mental health [35]. When the biomedical workforce is diversified, researchers will likely draw upon a broader array of healthcare frameworks, research questions [36–38], methodologies, and participant recruitment techniques that can overcome many barriers to health equity for at-risk communities [39–42]. Researchers of color are also more likely to support the equitable treatment of patients through the recognition of subtle patient cues, varying responses to clinical protocols, cultural responsiveness in increasing patient compliance, and a variety of other practice-oriented variables that would also lead to health equity [43, 44]. BUILD PODER employs CRT to provide linkages between research and social justice so that students can plainly see that their efforts to work on behalf of health equity will be feasible and meaningful. For example, in our first year, BUILD PODER students and PIs took a Toxic Tour of Los Angeles, learning about the unequal distribution of toxic waste based on income and race, then linking pollution to health disparities and the effects of toxic racism (http://www.cbecal.org/get-involved/toxic-tours/) [45]. The Health Equity Research and Education (HERE) Center, described below, will serve as a sustainable hub for health equity research.

### Barriers and educational inequities for students in context

Of particular concern are the many barriers, such as racism, to recruiting minorities into biomedical graduate programs [46] and optimizing their careers [47]. Education disparities in U.S. higher education have been documented for more than 40 years [48, 49] and show that racially and ethnically diverse students are especially underrepresented in science. For example, the 2017 National Center for Science and Engineering Statistics (NCSES) data reflect that while Latinos are 17.4% of the U.S. population, Latinos comprise only 11.5% of STEM bachelor’s degree recipients, 4.2% of STEM PhD recipients, and 4.5% of STEM faculty members in U.S. 4-year colleges and universities [13, 50]. In 2014, African Americans and Latinos earned 4.1% and 4.8%, respectively, a combined 8.9%, of all doctorates in science fields, much less than their collective representation in the general U.S. population [51]. There are many voices missing in biomedical research.

Despite efforts to close the achievement gap, barriers for traditionally underrepresented groups (URGs) are reflected in: (1) the length of time to graduation [52]; (2) lack of information or ambiguity in program requirements, guidelines, and requisite paperwork [53]; (3) feelings of isolation or absence of community [54]; (4) disappointment with the learning experience, lack of financial support, and lack of support from faculty/peers [53, 54]; and (5) lack of advising [55]. In a request for information, the NIH [46] found that the most salient issue among respondents was pipeline transition points. BUILD PODER addresses many of the barriers and transitions students face while becoming professionals without giving up one’s social identity [9, 20, 32].

Even when they successfully negotiate graduate degrees, students of color frequently feel alienated [56], required to live in a White world [49, 57], silenced and invisible [12, 20, 58], and are less likely to connect with a mentor [56, 59–61]. Critical Race Theory bridges theory and practice (praxis) by critically examining local and broader structures for inclusiveness and equity, by providing a counterspace for a conscious community of mentors and students [62] who share a framework that overturns the majoritarian narrative [18]. In honoring many voices, we hold high expectations, and provide challenging and meaningful research experiences in a supportive, “wise mentoring” training environment [63].

### BUILD PODER in local context

California State University, Northridge (CSUN) is one of 23 CSU campuses and, with 39,900 students, is a Minority Serving (MSI; 51.3% minority), a Hispanic Serving (HSI; 46% Latina/o), and an Asian American Native American Pacific Islander Serving (AANAPISI; 11% Asian/Asian American) Institution. CSUN has long been recognized by NSF’s Survey of Earned Doctorates as providing students with experiences and opportunities that put many of its STEM programs in the top 20 in the nation for matriculating undergraduates who go on to earn a doctorate in sciences, social sciences, and specifically in psychology (rated No. 1 among 529 comprehensive universities in recent years) [64]. Therefore, we have a large number and proportion of traditionally underrepresented students who go on to earn graduate degrees and secure meaningful careers, especially in the biomedical sciences. Each year, CSUN sponsors more than 1000 undergraduates in funded sponsored research experiences, with hundreds of other students benefiting from research laboratory and academic experiences. A recent survey from CSUN’s Office of Institutional Research found that 18% of seniors reported they have had a research experience while an undergraduate at CSUN. Of the primary NIH programs, Minority Access to Research Careers (MARC) and Career Opportunities in Research (COR) each reported approximately 50% of their students have gone on to a doctorate [65]. Nevertheless, data on CSUN undergraduate graduation rates suggest a persistent and significant lag in graduation rates for African American and Latina/o students. The gap for undergraduates is persistent, with traditionally URGs graduating at a rate 14.9% below their White counterparts for the 2009 cohort of freshmen.

### Linkages between activities and critical race theory tenets

In Table 1, we describe examples of how CRT is translated into program activities and the objectives that serve as goals for evaluation; each of which is based a tenet of the theory [3]. This table and our overall model, and two sample activities and outcomes for each tenet are intended to orient the reader to the implementation of the overall paradigm.

**Table 1:** Activities and Outcomes Related to the Tenets of Critical Race Theory. The 5 tenets of Critical Race Theory from Solorzano, Villalpando, and Oseguera, 2005 and their implications for BUILD PODER activities and outcomes

CRT Tenet: Centrality of Race and Racism	
“CRT acknowledges as its most basic premise that race and racism are defining characteristics of American society. In American higher education, race and racism are imbedded in the structures, practices, and discourses that guide the daily practices of universities.” *(P. 274)*	
Sample Activities	Outcome/Objective
• Student and mentor training; readings; field trips; film nights; fall conference; historical and structural perspectives on race, colorblindness, and historical trauma • New courses in CRT: Race, Racism, and Science; Public Health for Social Justice	• Awareness of historical and structural racism; preservation of native identities; new methods, theories • Sustainable structure for introducing students to STEM and CRT, linking research to social justice, contextualizing race/ethnicity in the sciences
CRT Tenet: Challenge to Dominant Ideologies	
“CRT in higher education challenges the traditional claims of meritocracy, objectivity, colorblindness, race neutrality, and equal opportunity.” *(P. 275)*	
Sample Activities	Outcome/Objective
• *Jumpstart* Summer program provides education, activities, and specific methods of countering racism in academe • Emphasis on mixed methods: Courses in qualitative methods; QuantCrit perspectives; faculty course in mixed methods research	• Students develop strategies for responding to racism through belonging, ownership, and empowerment • Innovative research; publish papers and write grants with new research questions and methods that are valid to the communities studied
CRT Tenet: Interdisciplinary	
“CRT challenges ahistoricism and the unidisciplinary focus of most analyses in educational research. In the field of higher education, this framework analyzes race and racism in both a historical and a contemporary context using interdisciplinary methods.” *(P. 275)*	
Sample Activities	Outcome/Objective
• ~100 faculty mentors, 5 community college partners, 5 research partners, 22+ departments • *Scholarship*: Tech tool for connecting faculty and students with one another and with grant opportunities, sharing equipment, expertise • Faculty Scholar Academies: Interdisciplinary mentored grant group; NRMN STAR grant-writing/coaching • Cluster Hires: Thematic hires inherently building interdisciplinary links in health equity across the Colleges of Health and Human Development and Social and Behavioral Sciences	• Multiple options for students; match is a good fit between student and mentor • Increased interdisciplinary grant proposals; new faculty connections, better communication, shared resources • Increased interdisciplinary grant proposals; new faculty connections, higher-level (R01) grants written • Thematic collaborations; greater grant and publication productivity; catalyzing new academe-community research partnerships, greater collaboration with research partners
CRT Tenet: Experiential	
“The application of a CRT framework in the field of higher education requires that the experiential knowledge of people of color be centered and viewed as a resource stemming directly from their lived experiences.” (*P. 275)*	
Sample Activities	Outcome/Objective
• Bi-weekly meetings and professional development courses with program director that include discussions of students’ backgrounds, holistic health, researcher identity, science as a profession, matching skills and career • BUILD Conferences: Fall - large audience, livestreamed to partners and community with presenters including Octavio Villalpando, Tim Wise, Karina Walters; and presenting research; Spring – campus-wide research competition	• Students have a sense of belonging and commitment to their native identity; students have tiered mentors and strategies for meeting academic challenges; seniors mentor juniors and sophomores; K-12 student projects, speakers as role models, opening possibilities • Connections among community members; morale and collaborations around race and racism; public dissemination of tenets; university-level discussions; policy changes
CRT Tenet: Commitment to Social Justice	
“In higher education, these theoretical frameworks are conceived as a social justice agenda that struggles to eliminate all forms of racial, gender, language, generation status, and class subordination.” *(P. 275)*	
Sample Activities	Outcome/Objective
• BUILD PODER and the Health Equity Research and Education (HERE) center sponsor research projects that address community-based needs, nonprofits, community clinics, health equity • Senior BUILD PODER Project: Community partnership with 4th, 8th, 12th grade classes around research	• Collaborative research and action grants around academe-community social problems and health equity solutions; publications and grants with social justice themes • Empirical study of research identity development; action research for BUILD PODER trainees; students giving back

## Student training aims, CRT use, and implementation

In order to increase the number of traditionally underserved students who pursue graduate degrees in the biomedical sciences, BUILD PODER was designed to focus on supporting and empowering the *whole* student, not just fill in gaps in their knowledge (i.e., increase research competencies). While it was essential for students to acquire social and cultural capital needed to succeed in academia, it was equally important for students to recognize that they come from communities of cultural wealth [66]. Through faculty mentorship, students gain important research skills in their respective fields and gain knowledge of what they need to get into graduate programs. Unlike traditional mentoring programs, our mentors undergo lengthy training to be culturally responsive to diverse students, increasing their mentoring effectiveness. Students from traditionally URGs are lost along the academic pipeline, often for reasons unrelated to grades or research skills. All facets of the student training are focused on empowering students to persist in science majors, gain access and thrive in graduate programs and in research careers. The curriculum helps students understand institutional policies and practices that may prevent them from persisting in higher education, learn to become their own advocates, and successfully confront social barriers and instances of inequities and discrimination.

### Using CRT in preparing students for biomedical careers

**Fig. 1:**
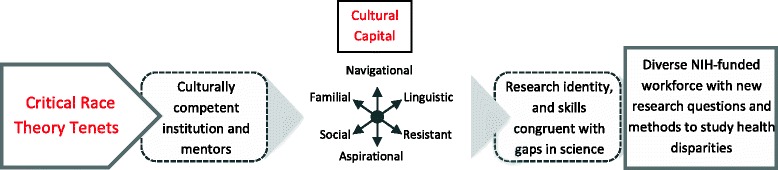
BUILD PODER Model. With Critical Race Theory as a foundation, we aim to transform our institution and mentors to recognize and integrate students’ cultural capital so that students develop a research identity and skills that will prepare them to develop novel approaches, methods, and interpretations as biomedical researchers who study and work toward health equity

Solórzano and Yosso [18] describe using critical race methodology as a theoretically grounded means of empowering students to engage their personal and cultural strengths or capital. Figure 1 depicts our program theory with desired outcomes. CRT tenets guide all aspects of the research training curriculum for undergraduate students as well as faculty development activity – please see Table 2.

**Table 2:** Student Programmatic Elements. Student activities and Critical Race Theory principles as applied in BUILD PODER

Summer JumpStart (SJS)	• Centrality of Race/Racism • Challenge to Dominant Ideologies • Interdisciplinary • Experiential Knowledge • Commitment to Social Justice	All newly accepted students attend a four-week intensive research program to help students transition to the culture of research that focuses on six elements: (1) hands-on faculty mentored research experience, (2) research ethics, (3) mentor-mentee relationships, (4) diversity training and empowerment, (5) community-building, including creating a peer supportive network, and (6) physical and mental well-being
Research Ethics Training	• Centrality of Race and Racism • Commitment to Social Justice	Students complete biomedical research training through CITI Program. In addition, discussions about health equity and unethical practices in science and in clinical settings enhance the online curriculum.
Faculty Mentored Research Experience	• Experiential Knowledge—draw on students’ capital: Navigational, Aspirational, Social, Linguistic, Resistant, Familial	Students learn to navigate the culture of research by working in a faculty mentored research lab; meet weekly with their mentors. Individual mentor-mentee meetings center on providing students social capital to reach their programmatic requirements and professional and academic preparation for graduate studies.
Summer Research Experience at Research Partners	• Experiential Knowledge—Navigational, Aspirational, Social, Linguistic, Resistant, and Familial capitals	We partnered with five doctoral granting institutions to provide students an intensive eight-week summer research experience. Students seek out potential mentors at these institutions or can apply to existing summer research experience programs. It is for many students their first time attending a predominantly white institution (PWI). This opportunity strengthens students’ research competencies and scientist identities.
Attending and Presenting at Professional Conferences	• Interdisciplinary • Experiential	In their first year, students learn the culture of research practices by presenting their research at a campus research symposium and are encouraged to attend and present at professional conferences in their field. Mentors accompany students to guide them and facilitate networking opportunities.
Weekly Community Meetings	• Centrality of Race/Racism • Challenge to Dominant Ideologies • Interdisciplinary • Experiential • Commitment to Social Justice	During the academic year, students meet weekly with the Student Training Program Director to address programmatic issues and to announce funding opportunities or upcoming events. The 1½-hour meetings also serve as a way to stay connected as a community, continue to address CRT-related issues during these meetings, plan for upcoming events, present guest speakers, and discuss issues and concerns that may arise.
Rigorous Coursework, Professional Development, Grantsmanship and Scholarships	• Centrality of Race/Racism • Challenge to Dominant Ideologies • Interdisciplinary • Experiential • Commitment to Social Justice	Students enroll in advanced research methods courses in their majors in preparation for graduate studies; departments without courses can develop curriculum. Students also enroll in two one-unit courses, *Getting into Graduate School* and *Surviving Graduate School*, to learn what to expect in graduate studies. In addition, students complete mock applications to fellowships and NIH-funded grants. Seniors enroll in a seminar where they collaborate on a science project with students in 4th, 8th, and 12th grade guided by CRT to “pay it forward.” In collaboration with the NIH, a series of student-centered grantsmanship webinars will broker the culture of research and funding students’ careers.

#### Centrality and intersection of race and racism

Throughout the year, discussions focus on issues of race, racism, sexism, and other forms of discrimination. Students are introduced to the research on gender and racial microaggressions [67], the influence of implicit biases and prejudice on interactions [68–70], impostor phenomenon [71–73], and stereotype threat [74]. Students are prepared to recognize microaggressions, name and effectively address them, thereby empowering students to affirm their identities as scientists of color and increase their persistence rates in academic settings. For example, many Latinos, in particular, begin their college careers at community colleges [75–77], some in part due to being advised by high school counselors to attend community colleges rather than 4-year schools. However, low transfer rates from community colleges prevent many low-income, predominantly ethnic minority students from reaching their academic goals [78]. BUILD PODER views community colleges as an untapped pool of potential scientists and essential to any recruitment efforts aimed at equity for Latinos; therefore the inclusion of community college students from partner institutions in the program is essential to creating a transfer-receptive culture at CSUN.

#### Centrality of students’ experiential knowledge

In an invited talk at the Association for Psychological Sciences in 2015, psychologist Robert Sellers stressed the importance of diversity in science, but cautioned that scientists must not discount URGs’ unique viewpoints and experiences. He stated, “If we’re only training people to think like us, then we’ve lost our advantage.” Yosso’s cultural community wealth [66] builds on students’ unique viewpoints, experiences and knowledge to integrate students’ language (linguistic capital), families (familial capital), and culture (cultural capital) with their science learning. BUILD PODER also focuses on students’ agency (navigational capital) in their academic success and treats their everyday discourse practices as fertile ground for academic learning [79]. Students are encouraged to maintain their cultural identities and use their community cultural wealth to think outside the box to solve complex science problems.

### Recruitment

Recruitment efforts focus on the visibility of BUILD PODER through personal contact, digital, social, and traditional media (e.g., fliers, postcards, brochures) at CSUN, community college partner campuses, and in the community. Beginning early in the academic year, staff make classroom announcements and attend school-wide events. Partnerships with advisement offices, the Educational Opportunity Program (EOP), and CSUN’s public relations office are essential. BUILD PODER hosts information sessions in the Fall semester, followed by application workshops that help to increase the number and quality of complete applications. The online application system allows us to track students’ application progress, enabling us to contact students who haven’t completed their applications. Last, there is no better recruitment tool than the students. The first Wednesday of every month is BUILD PODER Wednesday, when all students and program staff proudly wear their program t-shirts.

### Digital media

BUILD PODER uses its website (http://www.csun.edu/build-poder) to communicate with current and prospective students and faculty mentors and institutional partners. BUILD PODER has a “spotlight” section that highlights student accomplishments. The weekly e-Newsletter keeps the community up to date on upcoming events, and CSUN’s on-campus MIND screen (Matador Information Network Displays) televisions display current information about program events along with application information. BUILD PODER has also been featured twice in CSUN Today, a student operated e-magazine (http://csunshinetoday.csun.edu/education/22-million-awarded-to-csuns-build-poder-program/ and http://csunshinetoday.csun.edu/education/build-poder-welcomes-applicants-for-second-cohort-by-feb-5-deadline/).

### Social media

BUILD PODER has a Communications staff member who oversees the Facebook, Instagram, and Twitter accounts and posts BUILD PODER events, conferences, application workshops, and information sessions on social media. Posts also include relevant peer-reviewed articles and professional conference information. As much as social media is about getting information to people, it is also about engaging with other institutional entities at CSUN, our Pipeline and Research Partners, and the community.

## Supporting students by building faculty research and mentoring skills

BUILD PODER supports undergraduate scholars by (1) enhancing faculty research skills, (2) improving faculty mentoring competencies using the tenets of CRT, and (3) increasing campus-wide awareness and generating new practices around the tenets of CRT. When faculty research is stronger, students benefit from higher-quality research experiences, increased research infrastructure, and participation in publications and grant proposals [6, 40, 80, 81]. BUILD PODER pilot projects, for example, expose students to analyses of health risks and disparities among Asian sub-groups; a contextual analysis of the gut microbiome and obesity in Latina/o youth; and antimicrobial peptides as a potential solution for antibiotic resistance.

Incorporating the tenets of CRT into mentor training and competence, detailed below, opens up the consideration of structural racism as a barrier for students, faculty, universities, and science, and honors students’ experiential knowledge and motivation to generate social change, particularly in areas where health disparities exist [20, 80, 82–85].

BUILD PODER has initiated a gradual campus cultural transformation through a campaign of awareness, deconstruction, reconstruction, and praxis around racism [86]. BUILD PODER examines and deconstructs historical, institutional, and individual contributions to racism through large-scale, live-streamed conferences, speakers, and workshops on CRT, White privilege, and racism and science. Reconstruction and praxis take place through the integration of CRT tenets into existing university structures including curricular initiatives, the Graduation Initiative 2025 of the California State University, exploration of retention, tenure, and promotion criteria around mentoring [87], and coordination with EOP, faculty affairs, student affairs, and other campus entities.

### Faculty-student relationships in context

Faculty members at comprehensive institutions, typically academically successful and holding greater privilege than their students, often have a view of academia that differs from that of their students [88–92]. Trained in the same racist structures that built much of higher education, faculty members’ colorblindness and privilege contribute to students’ alienation and resistance [56, 84, 93, 94]. Rather than encouraging assimilation, faculty members can encourage resistance to stereotypes by (1) holding high standards while preparing and supporting students to reach higher in their academic experiences and goals (“wise mentoring” [63], (2) challenging negative group stereotypes and developing a healthy ethnic identity [9, 31], and (3) supporting positive racial/ethnic and science identities that honor experiential knowledge without reliance on “social construction of merit” [20, 32] that favors students who are already privileged [23].

### Mentor training through a critical race theory lens

Underlying much of the chasm between faculty and students are implicit structural and interpersonal sources of bias. While we know that having experience with others who are culturally different can make one more predisposed to want to work further with culturally different people [10, 95–98], mere exposure is not enough and tends to reinforce stereotypes due to concepts drawn from abstractions and symbolic racism, rather than personal interactions [99–101]. Neither students nor mentors receive adequate training to understand the psychosocial forces that shape the identities of the researchers [102–104]. Multicultural and diversity training have not been sufficient in making social and structural changes leading to equity; a new form of critical consciousness is needed to build a bridge between individuals from dominant and subordinate groups [10, 96, 105, 106]. “Cultural competence is more than the acquisition of knowledge and skills and must deal with hidden biases and prejudices” [106].

The application of CRT for faculty members starts with a 2-4 day, 16-h mentor training workshop that serves as a “soft” introduction to the theory and its application to biomedical research mentoring. In Year 1, faculty mentors learn to recognize their privilege and unconscious biases that lead them to fall prey to microaggressions and stereotype threat, to practice listening to students’ experiential knowledge and goals, and to engage in constructive dialogues about race, shown to increase mutual respect and understanding [107, 108]. In each of the two following years, 3- to 4-h follow-up training sessions, designed to strengthen one-on-one skills (Year 2) and to place CRT and its implications in historical and critical structural context to deepen mentor understanding of institutionalized and essentialized racism (Year 3) are required for all BUILD PODER mentors.

We commissioned a diversity and organizational change consulting firm who read our NIH BUILD proposal, read “Entering Mentoring” [109], and consulted with BUILD PIs about the role of CRT in training, discussing a vision for developing a new lens for biomedical mentor/mentee relationships based on CRT principles. Mentor training in the first year includes readings, lecture/discussion, role playing, case studies and other challenges to the race-based lenses we wear. As we worked with the leaders and trainers, the Four central topics are addressed in training: (1) Building Our Foundation for Cross-Cultural Mentoring Success, (2) Sticks and Stones May Break My Bones … But Words Can Hurt My Spirit, (3) Small Things That Send a Big Message: From Micro-Aggressions to Micro-Affirmations, and (4) When I Don’t Know I Don’t Know: Exploring Unconscious Bias and Privilege. These modules were intended to introduce increasingly controversial topics around race/ethnicity, privilege, and inequity. For example, faculty members are asked to evaluate their status privileges and those of their students to reflect upon the power dynamic in the research laboratory and to use “wise mentoring” [63] in which faculty learn to recognize a student’s strengths and hold high expectations while also assuring the student that she/he is capable of success on a given task.

## Infrastructure development: The health equity research and education Center

Colorblindness often translates into the superficial treatment of diversity through multiculturalism, a “race-neutral” approach to celebrating culture that ignores the structural and personal barriers that students of color face every day [5, 9, 23, 110]. Equity is not found in numbers. Where educational gaps between students of color and the general student body persist, HSI and AANAPISI institutions can be considered “Hispanic or AANAPISI-Enrolling” rather than “Hispanic or AANAPISI-Serving.” Indeed, diversity can be seen as a commodity for colleges and universities [17] who marginalize ethnic studies departments and allow or foster other departments to teach the content of ethnic studies departments, leading to further opportunities for the majoritarian voice to interpret the experiences of people of color without full comprehension of their lived experiences [18]. Even when the university makes changes to improve student services, these improvements often benefit the majority students the most, a form of interest convergence [17, 23, 111, 112].

Traditional views of diversity and equality justify the status quo while the players take on roles of collusion: White faculty show trepidation in working with others who are different from themselves [67, 83, 93]. Faculty of Color experience alienation and exhaustion [67, 83, 84, 113, 114]. Finally, a historically generated lack of awareness of the stressors and discrimination faced by People of Color at a large institution [82, 85] contributes to the majoritarian narrative of meritocracy and interest convergence.

**Fig. 2:**
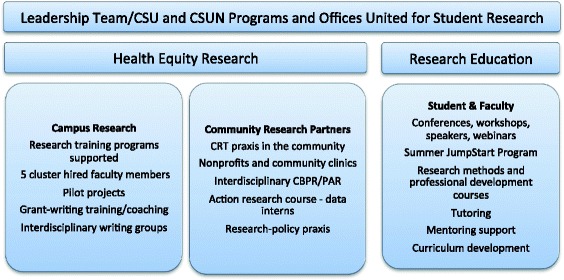
Health Equity and Research Education (HERE) Center Structure. HERE Center activities include campus and community research as well as research education

Cultural and institutional change through a CRT lens in a historically Predominantly White Institution (PWI) requires long-term planning and problem-solving across units. With the goal of intentionally centering students, faculty, and research topics that are traditionally marginalized, BUILD PODER’s Health Equity Research and Education (HERE) center will provide a home base and resource center for health equity research and researchers. Our center, sustained by external funding from training and research grants, will coordinate with many campus offices and programs including workforce diversity, community welfare, ethnic studies departments, Education, EOP, the Civil Discourse and Social Justice group, Deaf Studies, Student and Faculty Affairs, Institutional Research, and existing research training programs. HERE will support and synthesize campus and community-based research in health equity and train newer researchers (students and faculty members) to conduct socially relevant, interdisciplinary research to ultimately move to R01 competencies among faculty members whose interest is in social justice and health equity. Figure 2 shows the structure and activities for the HERE Center.

### Health equity research and education (HERE) Center goals

The overarching goals of the HERE center are to (1) increase grant activity in the area of health equity, (2) increase interdisciplinary and collaborative research in health equity, (3) increase community engagement and collaboration around health equity using Community Based, Participatory Research (CBPR) and Participatory Action Research (PAR), (4) increase health equity in the San Fernando Valley region, and (5) provide a focal point for biomedical research by centralizing campus training and resources for students, faculty, and community members related to health equity research and training.

### HERE leadership and framework

Sharing the CRT framework of BUILD PODER, the leadership of HERE will be largely based on group consensus and will be built on recommendations from BUILD PODER’s Local Steering Committee, which includes two NIH support officials and two experts in CRT and program implementation as well as two CRT consultants who work on program sustainability [3, 18, 67, 115–117]. Finally, BUILD PODER has a CRT Advisory Group with active members from several departments at CSUN who plan and support activities and are becoming increasingly involved in institutionalizing the tenets of CRT in curriculum initiatives such as the CSU’s Graduation Initiative 2025 and the development and implementation of BUILD PODER’s third mentor training module, planning the Fall conference, and writing a course on Race, Racism, and Science, ensuring that we are attracting the talent who may be drawn to biomedical sciences through a social justice agenda.

### HERE as a physical space

California State University, Northridge recently broke ground on “Research 1,” a new building, 10,000 square feet that will be occupied by two sets of cluster hires, one sponsored by BUILD PODER. In addition, Research 1 will serve as the physical home base for HERE students and researchers, BUILD PODER’s Health Equity Research and Education (HERE) center will house a student work space alongside a tutoring office, community college “transfer receptive” office, and resource center for undergraduate biomedical health equity research. HERE will also house three interdisciplinary cluster hires in health equity across the Colleges of Health and Human Development and Social and Behavioral Sciences. Several activities, highlighted in Fig. 2, provide a variety of services and opportunities that will build CSUN’s community partnerships and research capacity.

### HERE cluster hires

Three cluster faculty hires, followed by two additional faculty members within the next 3-5 years, will form a research group who will work on “Family Health and Disability Equity” (the cluster’s theme) by studying disparities in family-level resources, practices, and outcomes by racial/ethnic and social class status in the Los Angeles region. Engaging in this type of hiring will provide CSUN the opportunity to build upon existing strengths and spawn new areas of scholarship across campus by: enhancing the existing critical mass of faculty; promoting new and promising avenues of scholarship; meeting regional and national areas of need; growing interdisciplinary scholarship and collaboration; developing interdisciplinary curricula programs, and/or infrastructure to support student success; realizing institutional aspirations (e.g., diversifying faculty) and achieving campus priorities (e.g., growing scholarship and externally funded research); building community across the faculty and establishing collaboration as a value at CSUN; and serving as a mechanism for faculty retention and support for new faculty hires [118].

### The *Scholarship* app: Connecting researchers at CSUN

Building community among busy scholars is made easier through a newly created CSUN web platform, the “Faculty App” (www.csun.edu/faculty). The *Scholarship* tab on the App site is the home of this virtual network, which was created through partial funding from BUILD PODER. The overall goals of *Scholarship* are to create a stronger campus community, provide a platform for faculty to connect with fellow faculty, create more visibility for research projects, and encourage collaborative research. The key features include sections highlighting faculty research interests, projects, and resources, all searchable by other faculty, students, and the community.

### HERE data interns

An example of community health equity research partnerships is HERE’s “data interns,” well-trained statisticians and qualitative researchers who work with community nonprofits and health clinics. Data interns organize data, write codebooks, clean, screen, and analyze the data, and then prepare the data for potential evaluation or grant writing. HERE-sponsored students can conduct research with community nonprofits and clinics that will ultimately serve as the basis for community interventions, grant proposals, policy briefs, and publications. Already in place are community relationships and a course in Action Research that walks students through the theories, ethics, and processes of working with community-based organizations.

## Pipeline/research partnerships

Community colleges hold vast potential in diversifying the biomedical workforce [76, 119]. The community college is a common option for students of color: While 55% of Latina/o students, 42% of African American and 40% of Asian American students attend community college, only 36% of White students attend [120]. A study by the California Postsecondary Education Council (CPEC) found that only 17% of Latina/o community college students transferred to 4-year institutions in contrast to 41% of White and 39% of Asian American students [119]. This loss of talent can be ameliorated in part by providing students with a sense of belonging [121], a home base [122], and a transfer receptive culture [75]. Transfer receptive programs like BUILD PODER take into account that they, like community colleges, “must become relationship-centered institutions that focus on internal and external collaboration with all stakeholders” ([123, 124], p. 57).

BUILD PODER Pipeline Partners are four community colleges who host trainees at the sophomore level, with those students participating in the same activities as the students in the CSUN program on their local campus (Los Angeles Valley College, East Los Angeles College, Los Angeles Pierce College, Pasadena City College). To build community with students who are not on campus, community college students take part in bimonthly online meetings with PI Chavira and the Summer JumpStart Program, where high and consistent expectations are set for lab research with the goal of CSUN transfer. All Pipeline mentors participate in a four-hour CRT-based mentoring workshop. After struggling initially with student recruitment and building bridges between CSUN and campuses that are spread out over great geographic distances, we sought funds from a private foundation to recruit and support community college students. Trained at UCLA’s Center for Community College Partnerships, BUILD PODER’s new staff members are building relationships, starting newsletters, and participating in team-building activities across CSUN and Pipeline Partners.

Research partnerships with local University of California campuses and Claremont Graduate University provide both practical (research mentoring, coaching, providing information) and emotional or psychosocial support (empathizing, challenging, role modeling) [125, 126]. Research partners have welcomed BUILD PODER students as summer interns, integrating them into existing programs and building bridges with CSUN BUILD PODER students and faculty members. Research partners and community college partners have been invited to propose cross-campus collaborations that include BUILD PODER students to enhance research opportunities.

BUILD PODER is also building partnerships with local K-12 schools to support the early development of a research identity. McWilliam, Poronnik, & Taylor [127] have clear suggestions to correct for the “flight from science” that is well documented [128, 129]. Modifying science education to be active and creative forestalls boredom; “creativity is not the antithesis of scientific rigor but the core business of scientific thinking” [127, 130]. Among other outcomes, the program is intended to help BUILD PODER seniors learn how to break down the scientific process and “develop relevant skills and dispositions in and through dynamic team-oriented activity in which everyone shares the excitement of—and responsibility for—learning” [130]. BUILD PODER seniors design and K-12 students can select among 6 sessions in life sciences at grade-level with 4th, 8th, and 12th grade students at low-performing schools. Projects around health and health disparities are designed to awaken an interest in biomedical research based on the National Research Council’s Next Generation Science Standards [131]. Examples of projects at each grade level include food choice and health (4th grade level), air pollution (8th grade), and toxic landfills and water quality (12th grade). K-12 students present their work at the end of each Spring semester in a poster/paper forum.

## Site level evaluation design

Local site level evaluation for BUILD PODER is conducted in collaboration with the CSUN Center for Assessment, Research and Evaluation (CARE), the primary interface between CSUN activities and the Diversity Program Consortium (DPC) Coordination and Evaluation Center (CEC) at UCLA. CARE works to support BUILD PODER by coordinating evaluation activities at CSUN with those across the consortium, facilitating the collection of consortium-level data at CSUN, and conducting specific local site evaluation. Three core areas (Student Training, Research Enrichment, and Institutional) are examined at the local site level, each with its own specific aims and detailed evaluation plan.

### Student training

The overarching goal of evaluation activities focusing on student training is to examine changes in diversity, research preparation, and career development among CSUN students in the biomedical sciences. As Community College Pipeline Partners are central to the BUILD PODER program, the integration of students from partner colleges is a key component of evaluation activities. Of particular interest for the evaluation are the ways that the BUILD PODER program improves student understanding and experience in biomedical research (e.g. through the JumpStart program), enhances knowledge of CRT and research ethics, and serves to build a community among participating students. Unique elements of the evaluation approach include examining student perceptions of their experience of microaggressions and stereotype threat, how these perceptions affect their relationships with mentors, their development of a research identity, and overall interest in science and scientific research.

### Faculty development

Student-focused evaluation activities are complemented by faculty development. The overall evaluation objective is to assess the implementation and success of BUILD PODER in the following areas: (1) increasing and expanding the number of faculty engaged in research; (2) expanding the number of available training opportunities for research development and culturally sensitive mentoring; (3) growing the number of collaborative research opportunities; and (4) enhancing the number and quality of research programs and grant proposals. Particular attention is given to evaluating how innovations developed by BUILD PODER (e.g, Faculty Scholar Academies, and Culturally Competent Mentoring) work to enhance faculty engagement in research and grantsmanship, and promote student professional development in the biomedical sciences.

### Institutional development

The focus of the evaluation emphasizes specific aims related to the oversight, institutional development, and sustainability of the BUILD PODER program. Evaluation activities also assess infrastructure development at CSUN, opportunities for virtual collaborations, and new curriculum advancements, as well as the augmentation of external research partnerships with Pipeline colleges. Of specific interest are the transformations in the CSUN institutional culture and concomitant policies that emerge as a result of the implementation of BUILD PODER. As a unique component of BUILD PODER, particular attention is given to assessing the impact of the incorporation of principles of CRT at the university level as a guiding framework for promoting an inclusive and culturally sensitive culture for student and faculty research and mentoring.

#### Evaluation methods

Local site evaluation of the BUILD PODER program follows both ethnographic and quasi-experimental methods, and emphasizes both formative and summative evaluation. The specific evaluation methods vary depending on the aims of each core, and include: (1) pre- and post-test questionnaires; (2) analysis of existing data and documents; (3) individual and focus group interviews; and (4) ethnographic observations and virtual ethnographies. Formative evaluation activities utilize ethnographic observations and interviews to assess the quality and fidelity of implementation. Here, the CRT methodological framework is utilized to provide participants opportunities to express counter-stories that challenge dominant ideologies and to elicit multicultural affirmations. In order to assess summative outcomes, the evaluation of BUILD PODER employs pre- and post-test questionnaires as well as the analysis of existing data. Here, students and faculty are observed over time on domains related to research interest and engagement, professional research opportunities, mentor-mentee relationships, and experiences related to cultural bias and microaggressions.

## Conclusion: Potential contributions and challenges

### Unique features

#### CRT framework

CSUN’s BUILD PODER is one of two BUILD sites that have a focus on issues of race and discrimination, and is the only site that integrates structural, personal, and cultural factors through CRT and understanding the value of students’ social capital including cultural, linguistic, familial, and resistive strengths. While it is a process, developing a campus culture of openness and understanding of racism and its consequences allows for the potential to move beyond practices that reify educational inequity by exchanging ideas and developing new practices that are less hierarchical, more respectful, and open to ideas generated by students who often come from communities that experience health disparities. Students, faculty members, and administrators have been exposed to and have interacted with CRT concepts including racial/ethnic microaggressions, power relationships, and implicit bias, and are working toward models of laboratory relationships that honor and implement ideas generated by students in conjunction with their faculty mentors.

#### Anticipation and reduction of potential barriers to persistence

Awareness of factors contributing to low persistence among students from ethnic minority communities [64] has been crucial to finding resources to support students, aside from providing financial support (e.g., stipend, tuition remission). We coordinate with the financial aid office and have a “point person” whom students can ask for assistance; we secured priority registration so that students are able to register for all of their classes so they can graduate on time; and we provide one-on-one tutoring for gatekeeper courses in their major (e.g., calculus, organic chemistry, statistics) as well as provide writing coaches to strengthen their written assignments, essays, and manuscripts. We coordinate with the University Counseling Services (UCS) to provide stress management workshops during our weekly meetings. We are incorporating a well-being component, focusing on maintaining physical and mental health by coordinating with existing resources on campus such as the Wellness Center and department of kinesiology. We remain flexible about potential barriers and search for resources within our university.

#### Strength in unity

We have built a community of support and help students secure supportive networks within and outside of the university. Students know that they have a “home base” [122] if they encounter issues in graduate studies. They are not alone—they belong to a community. Evaluations from the first-year cohort have found that students have internalized this idea and it has strengthened their belief that they can succeed in graduate school.

#### Faculty and student complementary training

Whereas many undergraduate training programs take a deficit approach (focus on what students lack) in preparing students for graduate studies in the biomedical sciences, we acknowledge students’ strengths and we focus on strengthening both faculty mentors and students. Faculty mentors are trained to be culturally responsive, and students learn the skills to be successful in their fields through culturally competent training.

#### Challenges

There remain many challenges, including long-standing policies that reinforce inequities (e.g., late payment of financial aid requiring re-registration into less desirable courses), well-established resistance to personal reflection about one’s potentially racist beliefs and actions, and the sheer complexity of activities and tracking a large number of students (more than 100 each year). Several institutional variables have been addressed through programmatic arrangements with advisement, financial aid, and other entities. We continue, through annual training and continuous reinforcement of CRT concepts, to develop a campus culture that is critical of racial/ethnic bias and works actively toward making a change in education as a whole and in biomedical research specifically. One of the biggest challenges is recruitment of students and faculty mentors. At CSUN and at the Pipeline Partner institutions, students from traditionally underserved communities are less likely to apply to undergraduate research training programs because of low self-efficacy and a lack of identification as “biomedical,” a “scientist,” or a “researcher.” At the Pipeline Partner institutions, we have encountered additional challenges such as fewer students meeting the eligibility criteria due to part-time enrollment status, low grades in core science and math courses, familial obligations, and full-time work outside of school. In contrast to CSUN, where we have more than 100 faculty research mentors, we have only 18 research mentors at the Pipeline Partner institutions, due to part-time adjunct status, high teaching load, or a lack of facilities at the institution to conduct research. To address this challenge, in 2016 we were awarded a grant from the Annenberg Foundation to hire two recruitment specialists who will be primarily at the Pipeline institutions working with student services staff, faculty, and students in order to increase the pool of applicants and help recruit more research faculty mentors at these institutions.

The unique contributions of BUILD PODER lie in recognizing and working toward releasing structural constraints that influence personal goals by inculcating a sociological imagination [132], or the understanding that the structures that are influenced by racism, sexism, and other “isms” were built by humans, and can be understood and transformed to better meet the needs of the scientific enterprise.
